# Relationship between Secondhand Smoke Exposure and Depressive Symptoms: A Systematic Review and Dose–Response Meta-Analysis

**DOI:** 10.3390/ijerph16081356

**Published:** 2019-04-15

**Authors:** Changlin Han, Yangqun Liu, Xiao Gong, Xiaohua Ye, Junli Zhou

**Affiliations:** School of Public Health, Guangdong Pharmaceutical University, 283 Jianghai Dadao, Haizhu District, Guangzhou 510310, China; 13760851308@163.com (C.H.); yangqun_miemie@163.com (Y.L.); gongxiao@gdpu.edu.cn (X.G.)

**Keywords:** secondhand smoke, depressive symptoms, depression, epidemiology discipline

## Abstract

Previous studies have suggested an association between secondhand smoke (SHS) exposure and risk of depressive symptoms. However, it remains unclear whether there is a dose–response relationship. The effect estimates were pooled using fixed-effect or random-effect models based on homogeneity analysis. The dose–response meta-analysis was performed by linear and non-linear regression. Subgroup analyses were conducted to explore the possible sources of heterogeneity. Twenty-four studies were included in this meta-analysis. SHS exposure was significantly associated with increased odds of depressive symptoms (odds ratio (OR) = 1.32, 95% confidence interval (CI) 1.25–1.39). For SHS exposure expressed as an ordinal variable, the dose–response meta-analysis revealed a monotonically increasing relationship between SHS exposure and depressive symptoms. A similar dose–response relationship was observed for SHS exposure expressed as a continuous variable (OR = 1.57, 95% CI = 1.26–1.87). Our findings suggest that SHS exposure is associated with increasing odds of depressive symptoms in a dose–response manner.

## 1. Introduction

There is increasing evidence indicating that tobacco use causes several fatal diseases and also contributes to psychological problems [1,2]. Similarly, various psychiatric disorders had been reported to be associated with secondhand smoke (SHS) by cotinine and other tobacco-specific biomarkers [3,4]. It has well established that nicotine has impacts on the psychophysiological pathways through activating nicotinic acetylcholine receptors and causing intensive expression of nicotinic receptors in the central nervous system [3], which is associated with psychiatric disorders. In addition, scientific evidence has unequivocally established that inhaling SHS causes disease and death, and there is no risk-free level of SHS exposure [5,6]. It is conceivable that high levels of SHS exposure may cause or worsen psychological symptoms (e.g., anxiety and depressive disorders) as a result. Based on these results, we hypothesized that there may be a potential association between SHS exposure and depressive symptoms.

A significant body of studies have demonstrated the effects of SHS on the incidence of depressive symptoms [7,8,9,10]. Some of them reported significantly positive associations [7,8], while other studies revealed non-significant associations [9,10], indicating potential inconsistent results. So far only one meta-analysis reported that SHS exposure was significantly associated with depressive symptoms (OR = 1.60), but this study did not provide the information of dose–response association [11]. Therefore, it is still not clear whether there was a dose–response relationship between SHS exposure and depressive symptoms. In the present study, we performed a systematic search of observational studies on this issue, and then carried out dose–response meta-analyses to explore the potential dose–response relationship between SHS exposure and depressive symptoms.

## 2. Method

### 2.1. Search Strategy

The meta-analysis was conducted following the guidelines of preferred reporting items for systematic reviews and meta-analyses [12]. Studies were identified through a systematic literature search in the PUBMED and EMBASE electronic databases. The search details were conducted as follows: {“tobacco smoke pollution”(MeSH Terms) OR (“tobacco”(All Fields) AND “smoke”(All Fields) AND “pollution”(All Fields)) OR “tobacco smoke pollution”(All Fields) OR (“secondhand”(All Fields) AND “smoke”(All Fields)) OR “secondhand smoke”(All Fields)} AND {“depression”(MeSH Terms) OR “depression”(All Fields) OR (“depressive”(All Fields) AND “symptoms”(All Fields)) OR “depressive symptoms”(All Fields)}. In addition, reference lists of all the identified papers were reviewed for further eligible publications.

### 2.2. Inclusion and Exclusion Criteria

Two investigators (CL Han and YQ Liu) independently reviewed and assessed the eligibility of the identified studies using the following inclusion and exclusion criteria. The inclusion criteria required studies to: (1) use depressive symptoms as the outcome and use SHS as the exposure; (2) provide information assessing the association between SHS exposure and depressive symptoms; and (3) report original data including odds ratios (ORs) or relative risks (RRs) with corresponding 95% confidence internal (CI) (or information allowing us to compute them). Studies were excluded if they were not published as full reports, such as conference abstracts and letters to editors. When multiple reports were published on the same study population or subpopulation, only the most recent and informative study that met the inclusion criteria was included. Any discrepancies on whether an article merited inclusion between investigators were resolved by a consensus meeting of the three authors (CL Han, YQ Liu, and XH Ye).

### 2.3. Data Extraction

Two investigators (CL Han and YQ Liu) extracted data independently using a standardized form and then cross-checked the data together. Disagreements were resolved by consensus. For each study, the following characteristics were collected: first author’s name, publication year, geographical region, study design, age, sex, number of participants, diagnosis method of depressive symptoms, definition of SHS exposure, adjustment variables, and the adjusted ORs or RRs with their corresponding 95% CIs for the relation between SHS exposure and depressive symptoms.

### 2.4. Study Variables

The outcome variable was self-reported depressive symptoms measured by psychological scales (e.g., the Center for Epidemiologic Studies Depression scale (CES-D), the Patient Health Questionnaire-9 (PHQ-9), the Beck Depression Inventory questionnaire (BDI), the World Health Organization Global School-based Student Health Survey, the Edinburgh Postnatal Depression Scale, the Composite International Diagnostic Interview Short Form scales, the National Institute of Mental Health’s Diagnostic Interview Schedule for children Version IV, the Composite International Diagnostic Interview questionnaire), or unstructured questions. Unstructured questions mean that a few unstructured questions were used to measure depressive symptoms (e.g., “During the recent 12 months, have you ever felt sad or hopeless almost every day for two weeks in a row that you stopped doing some usual activities?”, or “In the past year, have you felt extremely sorrowful or despair for more than two weeks?”). The main independent variable was SHS exposure measured by self-report or biological tests. SHS exposure was expressed as binary (exposure versus non-exposure), continuous (days/week), or ordinal (e.g., none, 1–4 days/week, or ≥5 days/week) variables.

### 2.5. Quality Assessment

For each study retained for meta-analysis, the quality of study design was assessed by the forms for cross-sectional or prevalence study quality of an 11-item checklist [13]. An item would be scored “0” if it was answered “no” or “unclear”; if it was answered “yes”, then the item scored “1”. Article quality was assessed as follows: 0–3 for low quality, 4–7 for moderate quality, and 8–11 for high quality.

### 2.6. Statistical Analysis

All the statistical analyses were performed using Stata statistical software version 13.0 (StataCorp LP, College Station, Texas, USA). In general, a two-sided *p*-value of <0.05 was considered as being of statistical significance, except where specified. We generated forest plots for SHS exposure expressed as binary and continuous variables. All the effect estimates were pooled by either fixed-effect models using the method of Mantel and Haenszel (*p* for heterogeneity >0.1) or random-effect models using the method of DerSimonian and Laird (*p* for heterogeneity ≤0.1) [14,15]. Heterogeneity among studies was tested by the chi-squared test with the Cochrane *Q* statistic (significant at *p* ≤0.1) and quantified by *I*^2^ statistic [16]. In addition, subgroup analyses were performed according to geographical regions, study designs, SHS diagnoses, outcome diagnoses, types of psychological scales, sample sizes, excluding smokers, age groups, sex groups, SHS sources, and adjustment for covariates (e.g., social support, negative life events, and disease history), so as to explore the sources of heterogeneity. Disease history included any history of acute and chronic diseases (e.g., hypertension, diabetes, angina, congestive heart failure, coronary heart disease, heart attack, stroke, asthma, chronic bronchitis, cancer, or other diseases). Publication bias was evaluated by visual inspection of Begg’s funnel plot and tested by the Begg’s test (significant at *p* ≤ 0.1) [17].

To derive the dose–response relationship between SHS exposure and depressive symptoms, we carried out dose-response meta-analyses on frequency of SHS exposure expressed as ordinal variables. The dose-response meta-analyses were carried out using linear trend regression and restricted cubic spline regression, choosing the best-fitting model [18]. Firstly, we used a restricted cubic spline regression model with three knots to create spline variables, and then derived the generalized least squares trend estimation by including spline variables. Secondly, another linear regression model without the spline terms has also been fitted. Lastly, the significance of any non-linearity was examined by the likelihood ratio test that compared the model with the linear term only and the model with both the linear and the cubic spline terms. This analysis used information including the ORs and their corresponding 95% CI, number of cases and non-cases, and median of SHS exposure for each comparison group. For the open-ended upper interval of SHS exposure, we used 1.2-fold its lower limit as its midpoint [19]. When intervals of SHS exposure categories were reported, the midpoint of the interval was chosen.

## 3. Results

### 3.1. Characteristics of Studies

**Figure 1:**
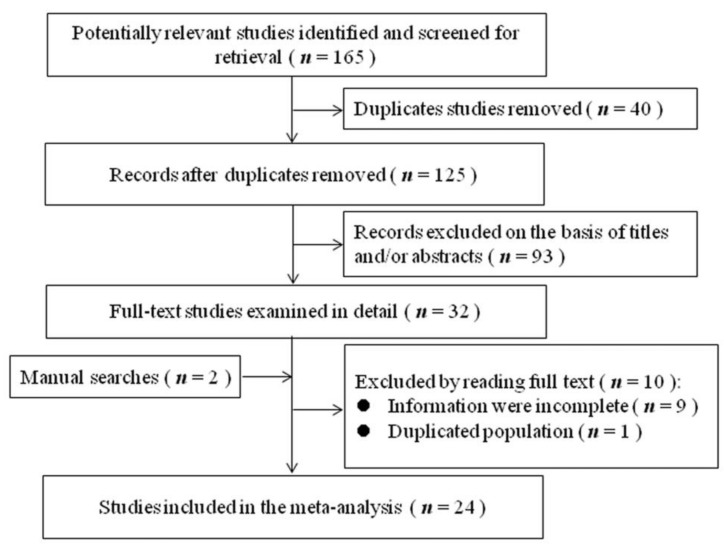
Flowchart of literature search and study selection.

The literature search and study selection process were shown in Figure 1. After excluding studies that did not meet the inclusion criteria, 24 studies were included in the present meta-analysis [7,8,9,10,20,21,22,23,24,25,26,27,28,29,30,31,32,33,34,35,36,37,38,39], including 22 cross-sectional studies and two cohort studies. All the study participants were from the healthy population, rather than the clinical population. The main characteristics and findings of these studies on SHS exposure and depressive symptoms are given in Table 1. Sixteen of these studies were conducted in Asia, seven were conducted in the United States (USA), and one was conducted in Europe. In terms of SHS exposure, 24 studies reported the binary exposure (exposure versus non-exposure) and 11 reported the frequency of exposure. The quality score of each study was presented in Table 1. Six studies were of high quality, 18 studies were of moderate quality, and no study was of low quality.

### 3.2. Relationship between Binary SHS Exposure and Depressive Symptoms

**Figure 2:**
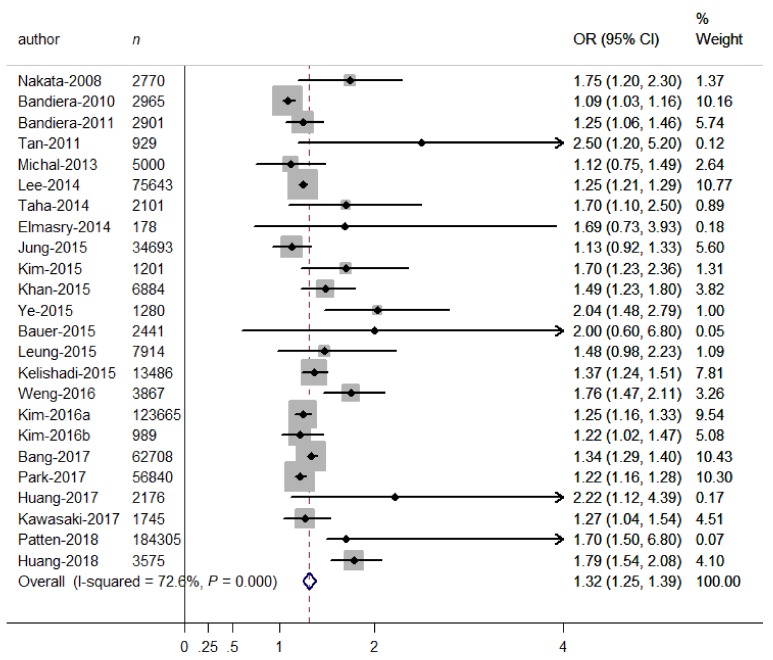
Forest plot for the association between binary SHS exposure (exposure vs. non-exposure) and depressive symptoms. Grey square represents the effect estimate in each study, with square size reflecting the study-specific weight and the 95% CI represented by horizontal bars. The diamond indicates the summary effect estimate.

The overall OR of depressive symptoms comparing SHS exposure with non-exposure was presented in Figure 2. An overall 32% increase in the odds of depressive symptoms was observed for SHS exposure (OR = 1.32, 95% CI 1.25–1.39), and some heterogeneity was observed (*I*^2^ = 72.6%, *p* < 0.001). Therefore, we carried out stratified analyses to assess the heterogeneity across subgroups (Table 2). As to SHS diagnoses, the relation was significantly stronger (*p* for differences between subgroups <0.001) in studies using self-reported SHS (OR = 1.34, 95% CI = 1.27–1.41) than in those using biological SHS (OR = 1.11, 95% CI = 1.04–1.17), indicating that using self-reported SHS may overestimate the adverse relation. As to outcome diagnoses, the relation was significantly stronger (*p* for differences between subgroups = 0.037) in studies using psychological scales (OR = 1.45, 95% CI = 1.29–1.61) than in those using unstructured questions (OR = 1.27, 95% CI = 1.21–1.32), indicating that using unstructured questions may underestimate the adverse relation. As to types of psychological scales, there were significant differences in OR estimates (OR = 1.67, 95% CI = 1.35–1.99, for CES-D scales; OR = 1.30, 95% CI = 1.09–1.51, for BDI scales; OR = 1.10, 95% CI = 1.03–1.16, for PHQ-9, OR = 1.29, 95% CI = 1.24–1.35, for others; *P* for differences between subgroups = 0.001), indicating that using the CES-D may overestimate the adverse relation. When doing subgroup analyses on adjustment for covariates, significant differences were observed between adjusted and unadjusted ORs for social support (OR = 1.84 versus OR = 1.28, *p* for differences between subgroups < 0.001), negative life events (OR = 1.67 versus OR = 1.28, *p* for differences between subgroups = 0.040) and disease history (OR = 1.37 versus OR = 1.25, *p* for differences between subgroups = 0.070), suggesting that non-adjustment for these covariates may underestimate the adverse relation. However, there were non-significant differences between subgroups according to study locations (*p* = 0.539), study designs (*p* = 0.524), sample sizes (*p* = 0.651), excluding smokers (*p* = 0.183), age groups (*p* = 0.394), sex groups (*p* = 0.370), SHS sources (*p* = 0.426), and adjustment for disease history (*p* = 0.070). No publication bias was observed from visual inspection of the funnel plot and from the Begg’s test (*p* = 0.385), indicating that SHS exposure was consistently associated with increased odds of depressive symptoms.

### 3.3. Relationship between SHS Exposure Expressed as a Continuous Variable and Depressive Symptoms

**Figure 3:**
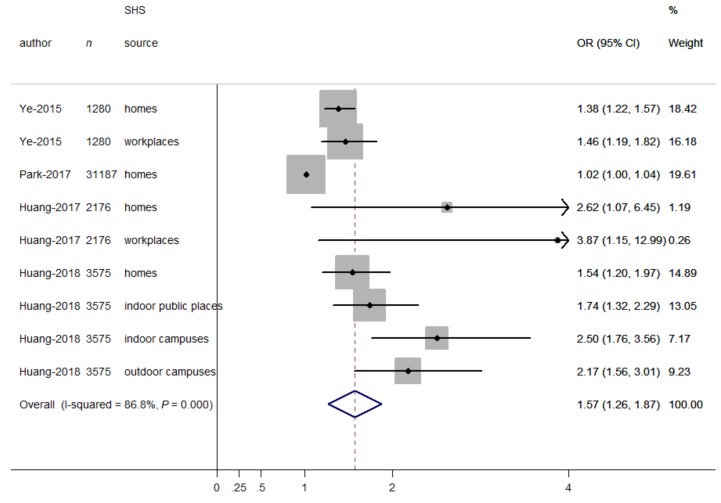
Forest plot for the association between SHS exposure (days/week) and depressive symptoms. Grey square represents the effect estimate in each study, with square size reflecting the study-specific weight and the 95% CI represented by horizontal bars. The diamond indicates the summary effect estimate.

When SHS exposure was expressed as a continuous variable (days/week), the relationship between continuous days of SHS exposure and depressive symptoms was presented in Figure 3. The overall OR for having depressive symptoms increased with increasing days of SHS exposure (OR = 1.57, 95% CI = 1.26–1.87), indicating that the odds for having depressive symptoms increased with every one-day increment in SHS exposure. In addition, some heterogeneity was observed (*I*^2^ = 86.8%, *p* < 0.001). Therefore, we carried out stratified analyses to assess the heterogeneity across subgroups defined by sex groups, age groups, and SHS sources. In terms of SHS sources, there were significant differences in the odds of depressive symptoms (OR = 2.30, 95% CI = 1.74–2.86, for campuses; OR = 1.74, 95% CI = 1.26–2.23, for public places; OR = 1.47, 95% CI = 1.15–1.78, for workplaces; OR = 1.30, 95% CI = 0.97–1.62, for homes; *p* for differences between subgroups = 0.019). However, there were non-significant differences between studies in different sex groups (OR = 1.00, 95% CI = 0.97–1.03, for males; OR = 1.22, 95% CI = 0.87–1.56, for females; *p* for differences between subgroups = 0.213) and age groups (OR = 1.39, 95% CI = 1.21–1.56, for adults; OR = 1.24, 95% CI = 0.74–1.75, for adolescents; *p* for differences between subgroups *=* 0.582). No publication bias was observed from visual inspection of the funnel plot and from the Begg’s test (*p* = 0.917), indicating that days of SHS exposure was consistently associated with increased odds of depressive symptoms.

### 3.4. Relationship between SHS Exposure Expressed as Ordinal Variables and Depressive Symptoms

**Figure 4:**
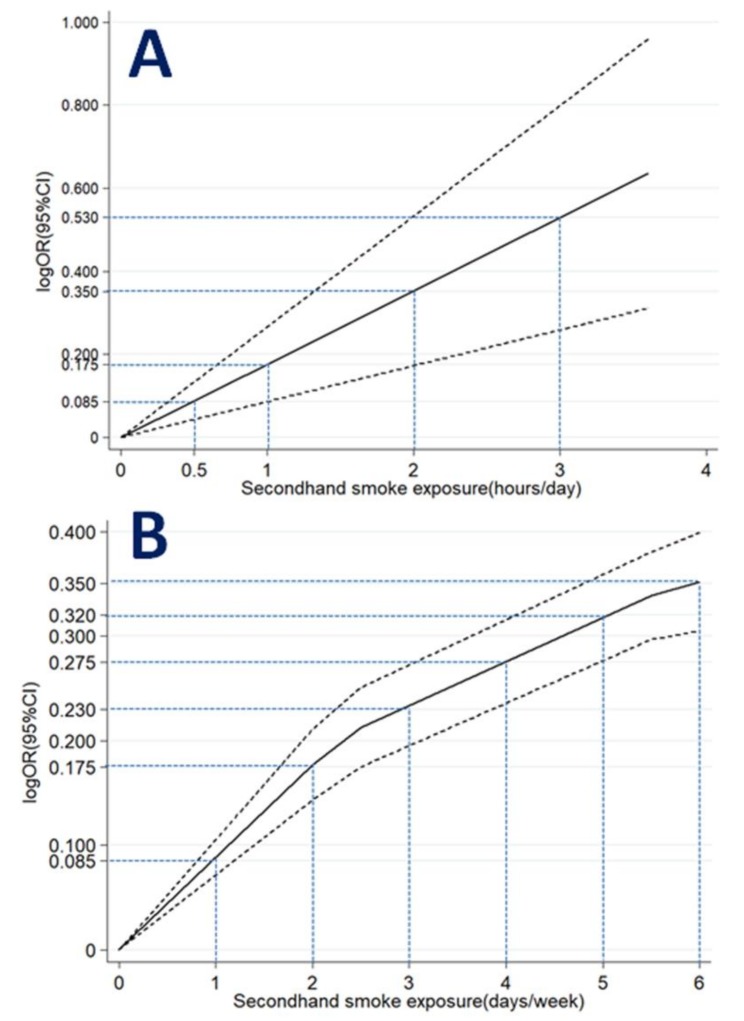
Association between SHS exposure (**A**) hours/day; (**B**) days/week and depressive symptoms obtained by dose–response meta-analyses. Solid line represents the estimated odds ratio and the dot-dashed lines represent the 95% confidence intervals.

When hours of SHS exposure per day were expressed as ordinal variables (Table 3), the dose–response meta-analysis model revealed a linear relationship between hours of SHS exposure and odds of depressive symptoms (*p* for linear trend <0.001; Figure 4A). A monotonically increasing relationship was consistently observed for hours of SHS exposure (OR = 1.09 for half-hour per day; OR = 1.19 for one hour per day; OR = 1.42 for two hours per day; OR = 1.70 for three hours per day).

When days of SHS exposure per week were expressed as ordinal variables (Table 4), the dose–response meta-analysis model revealed a non-linear relationship between days of SHS exposure and depressive symptoms (*p* for non-linear trend <0.001; Figure 4B). A monotonically increasing relationship was observed for days of SHS exposure (OR = 1.09 for one day per week; OR = 1.19 for two days per week; OR = 1.26 for three days per week; OR = 1.32 for four days per week; OR = 1.38 for five days per week; OR = 1.42 for six days per week).

## 4. Discussion

The present meta-analysis included more new studies to confirm the positive association between binary SHS exposure and depressive symptoms (OR = 1.32, 95% CI 1.25–1.39). In addition, this study contributes to the literature by finding a monotonically increasing dose–response relationship between SHS exposure expressed as an ordinal variable and odds of depressive symptoms. A similar dose–response relationship was observed for SHS exposure expressed as a continuous variable (OR = 1.57, 95% CI = 1.26–1.87).

Increasing epidemiological studies have focused on studying the potential association between SHS exposure and depressive symptoms, but the findings are inconsistent. For example, recent evidence revealed that SHS exposure was significantly associated with higher rates of depressive symptoms [7,8,24,29], but a non-significant relation was found in other studies [9,10]. Evidence from this updated meta-analysis of 24 observational studies revealed that an overall 32% increase in the odds of depressive symptoms was observed for SHS exposure (OR = 1.32, 95% CI 1.25–1.39). This finding is consistent with the only meta-analysis in the past, which only included seven observational studies and reported a 60% increase in the prevalence of depressive symptoms for SHS exposure (OR = 1.60, 95% CI 1.35–1.90) [11]. Notably, only two prospective studies were conducted in the past 10 years, which may reduce the power of revealing the association between SHS exposure and depressive symptoms. Therefore, more longitudinal studies are needed to address the causal link in the future. Notably, as to the relationships for SHS exposure expressed as a continuous variable (days/week) based on four studies, the stratified analysis revealed that there were significant differences in the OR estimates between subgroups according to SHS sources (*p* for differences between subgroups = 0.019). However, as to the relationships for binary SHS exposure based on 24 studies, the stratified analysis found that there were non-significant differences in the OR estimates between subgroups according to SHS sources (*p* = 0.426). The above inconsistent results on exposure-specific association may be due to differences in the number of included studies, measurement methods for SHS (self-report or cotinine level) and depressive symptoms (psychological scales or unstructured questions), prevalence rates of SHS exposure and depressive symptoms, and so on.

The most important question remains unclear, and that is the dose–response relationship between SHS exposure and depressive symptoms. Some recent studies revealed linear trends between SHS exposure and depressive symptoms [7,22,24], but non-significant associations were observed in other studies [25,31], suggesting that these findings are inconsistent. To the best of our knowledge, there is still no meta-analysis available to explore this dose–response relationship. A novel aspect of this study is to clarify the potential dose–response relationship, so a dose–response meta-analysis was performed for SHS exposure expressed as an ordinal variable. An important finding is that there is a linear relationship for hours of SHS exposure (*p* for linear trend < 0.001) and a non-linear relationship for days of SHS exposure (*p* for non-linear trend < 0.001), indicating a monotonically increasing trend for SHS exposure. Additionally, when SHS exposure was expressed as a continuous variable (days/week), the odds for having depressive symptoms increased with increasing days of SHS exposure (OR = 1.57, 95% CI = 1.26–1.87), which may provide more evidence for potential dose-response relationships. Notably, previous studies only focused on the linear model to explore above dose-response relationship, and none have reported these relationships based on non-linear models. Our findings suggest that future studies should focus on both linear and non-linear models to reveal the potential relationship.

Some methodological limitations and uncontrolled variables in the included studies may contribute to the heterogeneity between studies. First, most of the studies included in this meta-analysis used self-reported questionnaires instead of a more accurate biological measure to confirm SHS exposure, resulting in an underestimation or overestimation of the true association. Consistent with the previous meta-analysis [11], the subgroup analysis stratified by SHS diagnosis revealed that the adverse relation was significantly stronger in studies using self-reported SHS than in those using biological SHS, indicating that using self-reported SHS may overestimate the odds of depressive symptoms. However, for studies with the cross-sectional design, there is a possibility that study participants with higher levels of depressive symptoms may tend to report more SHS exposure. Therefore, results from this study need to be confirmed in future meta-analyses including more longitudinal studies. Second, although most of the depression scales have high reliability and validity [40,41,42], the different diagnosis methods for depressive symptoms applied in the studies may have resulted in biases in pooled estimates. Our subgroup analysis by outcome diagnosis indicated that the adverse association was significantly stronger in studies using psychological scales than in those using unstructured questions, indicating that using unstructured questions may underestimate the odds of depressive symptoms. Third, other covariates may have potential influences on effect estimates. Our subgroup analyses revealed that there were significant differences between adjusted and unadjusted ORs for social support and negative life events, suggesting that non-adjustment for these covariates may underestimate the association between SHS exposure and depressive symptoms.

There are several behavioral and biological mechanisms that may explain the relationship between SHS exposure and depressive symptoms. First, SHS exposure may be an indicator of stressful working and living environments, which in turn may lead to depressive symptoms or depression [43,44]. Second, SHS exposure has been associated with adverse health effects. All of the adverse effects (such as cancer, respiratory disease, and so on) in turn may cause depressive symptoms by direct and indirect multi-step process [6,45]. Third, SHS exposure may contribute to lowering levels of dopamine and γ-aminobutyric acid, which have been related to an increased risk for depressive symptoms as observed in firsthand smokers [46,47]. Finally, the adverse effect of SHS exposure has been attributed to the inflammation-associated mechanism. SHS exposure may induce the body to produce inflammatory cytokines, which can contribute directly to the development of depressive symptoms [48,49].

A novel aspect of this study is that the information on continuous and ordinal frequency of SHS exposure was considered in order to better understand the dose–response relationship. However, there are some potential limitations to this study. First, because of resource limitations, this study did not include some unpublished studies, which may bring some publication bias. However, the likelihood should be small, since the funnel plot and Begg’s test indicated no evidence of publication bias among all the studies included in this meta-analysis. Second, although it is very meaningful to explore the potential dose–response relationships on dose or duration of SHS exposure, there are not sufficient data on dose and duration of SHS exposure to carry out such dose–response meta-analyses. Third, a relatively small sample size of longitudinal studies may have limited our power of revealing the association between exposure and outcomes. Therefore, more longitudinal studies are needed to address their causality.

## 5. Conclusions

This meta-analysis confirms that SHS exposure is positively associated with odds of depressive symptoms. Additionally, this study adds to existing knowledge by finding linear and non-linear dose–response relationships between SHS exposure and depressive symptoms. These findings highlight the significance of reducing SHS exposure worldwide, and more longitudinal studies are needed to establish the causal relationship in the future.

## Figures and Tables

**Table 1:** Main characteristics of studies in the meta-analysis.

Author	Location	Design	Outcome Diagnosis	SHS Diagnosis	*n*	OR (95% CI)	Quality Score
Nakata-2008	Asia	CS	scale	BT	2770	1.75 (1.20–2.30)	6
Bandiera-2010	USA	CS	scale	BT	2965	1.09 (1.03–1.16)	6
Bandiera-2011	USA	CS	scale	BT	2901	1.25 (1.06–1.46)	7
Tan-2011	USA	CS	scale	SR	929	2.50 (1.20–5.20)	6
Michal-2013	Europe	CS	scale	SR	5000	1.12 (0.75–1.49)	6
Lee-2014	Asia	CS	unstructured question	SR	75,643	1.25 (1.21–1.29)	8
Taha-2014	USA	CS	scale	SR	2101	1.70 (1.10–2.50)	8
Elmasry-2014	USA	cohort	scale	SR	178	1.69 (0.73–3.93)	6
Jung-2015	Asia	CS	unstructured question	SR	34,693	1.13 (0.92–1.33)	7
Kim-2015	Asia	CS	scale	SR	1201	1.70 (1.23–2.36)	7
Khan-2015	USA	CS	unstructured question	SR	6884	1.49 (1.23–1.80)	6
Ye-2015	Asia	CS	scale	SR	1280	2.04 (1.48–2.79)	8
Bauer-2015	Asia	CS	scale	SR	2441	2.00 (0.60–6.80)	6
Leung-2015	Asia	cohort	scale	SR	7914	1.48 (0.98–2.23)	7
Kelishadi-2015	Asia	CS	scale	SR	13,486	1.37 (1.24–1.51)	7
Weng-2016	Asia	CS	scale	SR	3867	1.76 (1.47–2.11)	6
Kim-2016a	Asia	CS	unstructured question	SR	123,665	1.25 (1.16–1.33)	8
Kim-2016b	Asia	CS	scale	SR	989	1.22 (1.02–1.47)	8
Bang-2017	Asia	CS	unstructured question	BT	62,708	1.34 (1.29–1.40)	7
Park-2017	Asia	CS	unstructured question	SR	56,840	1.22 (1.16–1.28)	7
Huang-2017	Asia	CS	scale	SR	2176	2.22 (1.12–4.39)	8
Kawasaki-2017	Asia	CS	scale	SR	1745	1.27 (1.04–1.54)	7
Huang-2018	Asia	CS	scale	SR	3575	1.79 (1.54–2.08)	7
Patten-2018	USA	CS	scale	SR	184,305	1.70 (1.50–6.80)	7

Note: *n*, number of participants; OR, odds ratio; CS, cross-sectional; SHS, secondhand smoke; SR, self-reported; BT, biological test.

**Table 2:** Pooled ORs of depressive symptoms in strata of selected covariates.

Subgroups		No. of Studies	OR (95% CI)	Statistical Method	*p*-Value for Heterogeneity ^b^
All studies		24	1.32 (1.25–1.39)	random	
Location	Asia	16	1.33 (1.26–1.40)	random	0.539
	USA	7	1.30 (1.09–1.51)	random	
	Europe	1	1.12 (0.75–1.49)	fixed	
Study design	cross-sectional	22	1.32 (1.25–1.39)	random	0.524
	cohort	2	1.51 (0.93–2.09)	fixed	
SHS diagnosis	self-report	22	1.34 (1.27–1.41)	random	<0.001
	biological test	2	1.11 (1.04–1.17)	fixed	
Outcome diagnosis	psychological scale	18	1.45 (1.29–1.61)	random	0.037
	unstructured question	6	1.27 (1.21–1.32)	random	
Types of psychological scales	CES-D	6	1.67 (1.35–1.99)	random	<0.001
	PHQ-9	3	1.10 (1.03–1.16)	fixed	
	BDI	3	1.30 (1.09–1.51)	fixed	
	Others ^a^	12	1.29 (1.24–1.35)	random	
Sample size	>500	23	1.32 (1.25–1.39)	random	0.651
	≤500	1	1.69 (0.73–3.93)	fixed	
Excluding smokers	Yes	16	1.33 (1.23–1.42)	random	0.183
	No	8	1.26 (1.22–1.30)	random	
Age group	adolescent	9	1.31 (1.24–1.38)	random	0.394
	adult	14	1.38 (1.23–1.52)	random	
Sex group	female	14	1.28 (1.20–1.35)	random	0.370
	male	6	1.24 (1.20–1.29)	fixed	
SHS source	home	18	1.30 (1.24–1.35)	random	0.426
	workplace	5	1.53 (1.03–2.02)	random	
	public place	3	1.44 (1.26–1.63)	fixed	
	campus	2	1.32 (1.25–1.38)	random	
Adjustment for social support					
	yes	3	1.84 (1.59–2.08)	fixed	<0.001
	no	21	1.28 (1.22–1.34)	random	
Adjustment for negative life events					
	yes	6	1.67 (1.31–2.04)	random	0.040
	no	18	1.28 (1.21–1.36)	random	
Adjustment for disease history					
	yes	11	1.37 (1.24–1.49)	random	0.070
	no	13	1.25 (1.22–1.29)	fixed	

Notes: BDI, Beck Depression Inventory questionnaire; CES-D, Center for Epidemiologic Studies Depression scale; OR, odd ratios; PHQ-9, Patient Health Questionnaire-9; SHS, secondhand smoke; ^a^ Others included the World Health Organization Global School-based Student Health Survey, the Edinburgh Postnatal Depression Scale, the Composite International Diagnostic Interview Short Form scales, the National Institute of Mental Health’s Diagnostic Interview Schedule for children Version IV, the Composite International Diagnostic Interview questionnaire, or unstructured questions. ^b^ Chi-squared test was used to test the heterogeneity between subgroups.

**Table 3:** Epidemiological studies of frequency of SHS exposure (hours/day) and depressive symptoms.

Author	SHS Source	SHS Frequency (Hours/Day)	Midpoint Frequency (Hours/Day) ^a^	OR (95% CI)
Jung-2015 (male)	workplace	0	0	1.00
		<1	0.5	0.92 (0.77–1.09)
		≥1	1.2	1.23 (0.97–1.54)
Jung-2015 (female)	workplace	0	0	1.00
		<1	0.5	0.89 (0.76–1.04)
		≥1	1.2	1.32 (1.06–1.64)
Jung-2015 (male)	home	0	0	1.00
		<1	0.5	0.90 (0.65–1.23)
		≥1	1.2	1.21 (0.69–2.13)
Jung-2015 (female)	home	0	0	1.00
		<1	0.5	1.18 (1.02–1.35)
		≥1	1.2	1.71 (1.34–2.18)
Kim-2016 (male)	home	0	0	1.00
		<1	0.5	0.99 (0.66–1.48)
		1–2.9	2.0	2.01 (1.04–3.86)
		≥3	3.6	1.87 (1.43–2.44)
Kim-2016 (female)	home	0	0	1.00
		<1	0.5	0.98 (0.87–1.11)
		1–2.9	2.0	1.37 (1.07–1.75)
		≥3	3.6	1.56 (1.42–1.72)

Notes: OR, odds ratio; SHS, secondhand smoke; ^a^ When intervals of aspirin categories were reported, the midpoint of the interval was chosen; for the open-ended upper interval, we used 1.2-fold its lower limit.

**Table 4:** Epidemiological studies of frequency of SHS exposure (days/week) and depressive symptoms.

Author	SHS Source	SHS Frequency (Days/Week)	Midpoint Frequency (Days/Week) ^a^	OR (95% CI)
Lee-2015	home	0	0	1.00
		1–4	2.5	1.22 (1.17–1.27)
		≥5	6.0	1.36 (1.29–1.43)
Ye-2015	home	0	0	1.00
		1–3	2.0	2.12 (1.41–3.21)
		4–7	5.5	2.53 (1.70–3.78)
Ye-2015	workplace	0	0	1.00
		1–3	2.0	2.08 (1.25–3.45)
		4–7	5.5	2.58 (1.16–3.73)
Huang-2017	home	0	0	1.00
		1–3	2.0	1.73 (0.66–4.49)
		4–7	5.5	2.36 (1.09–5.13)
Huang-2017	workplace	0	0	1.00
		1–3	2.0	1.42 (0.31–6.54)
		4–7	5.5	3.19 (1.17–8.74)
Huang-2018	public place	0	0	1.00
		1–4	2.5	1.28 (1.06–1.53)
		5–7	6.0	1.66 (1.30–2.10)
Huang-2018	home	0	0	1.00
		1–4	2.5	0.98 (0.78–1.24)
		5–7	6.0	1.50 (1.22–1.85)
Huang-2018	indoor campus	0	0	1.00
		1–4	2.5	1.36 (1.08–1.71)
		5–7	6.0	2.13 (1.56–2.91)
Huang-2018	outdoor campus	0	0	1.00
		1–4	2.5	1.37 (1.11–1.68)
		5–7	6.0	1.83 (1.38–2.44)

Note: OR, odds ratio; SHS, secondhand smoke. ^a^ When intervals of aspirin categories were reported, the midpoint of the interval was chosen; for the open-ended upper interval, we used 1.2-fold its lower limit.
